# Corrigendum: Transcription Factors Tec1 and Tec2 Play Key Roles in the Hyphal Growth and Virulence of *Mucor lusitanicus* Through Increased Mitochondrial Oxidative Metabolism

**DOI:** 10.71150/jm.2504100

**Published:** 2025-04-29

**Authors:** Viridiana Alejandre‑Castaneda, J. Alberto Patino‑Medina, Marco I. Valle‑Maldonado, Alexis Garcia, Rafael Ortiz‑Alvarado, Leon F. Ruiz‑Herrera, Karla Viridiana Castro‑Cerritos, Joel Ramirez‑Emiliano, Martha I. Ramirez‑Diaz, Victoriano Garre, Soo Chan Lee, Victor Meza‑Carmen

**Affiliations:** 1 Instituto de Investigaciones Químico Biológicas, Universidad Michoacana de San Nicolás de Hidalgo, Ciudad Universitaria, 58030, Morelia, Mexico; 2 Laboratorio Estatal de Salud Pública del Estado de Michoacán, 58279, Morelia, Mexico; 3 Department of Molecular Microbiology and Immunology, South Texas Center for Emerging Infectious Diseases (STCEID), The University of Texas at San Antonio, San Antonio, 78249, USA; 4 Instituto de Química Aplicada, Universidad Papaloapan, Campus Tuxtepec, 68301, Tuxtepec, Mexico; 5 Departamento de Ciencias Médicas, Universidad de Guanajuato, 37320, León, Mexico; 6 Departamento de Genética y Microbiología, Facultad de Biología, Universidad de Murcia, 30100, Murcia, Spain; 7 Instituto de Investigaciones Químico Biológicas, Universidad Michoacana de San Nicolás de Hidalgo, Ciudad Universitaria, 58030, Morelia, Mexico

Published online : December 19, 2023

https://doi.org/10.1007/s12275-023-00096-8

­

After publication, there were detected 2 mistakes in the figures, 1 and 5 of the manuscript:

**Published figure 1**

You can see the photos duplicated by mistake in a pink boxes (Figure 1).

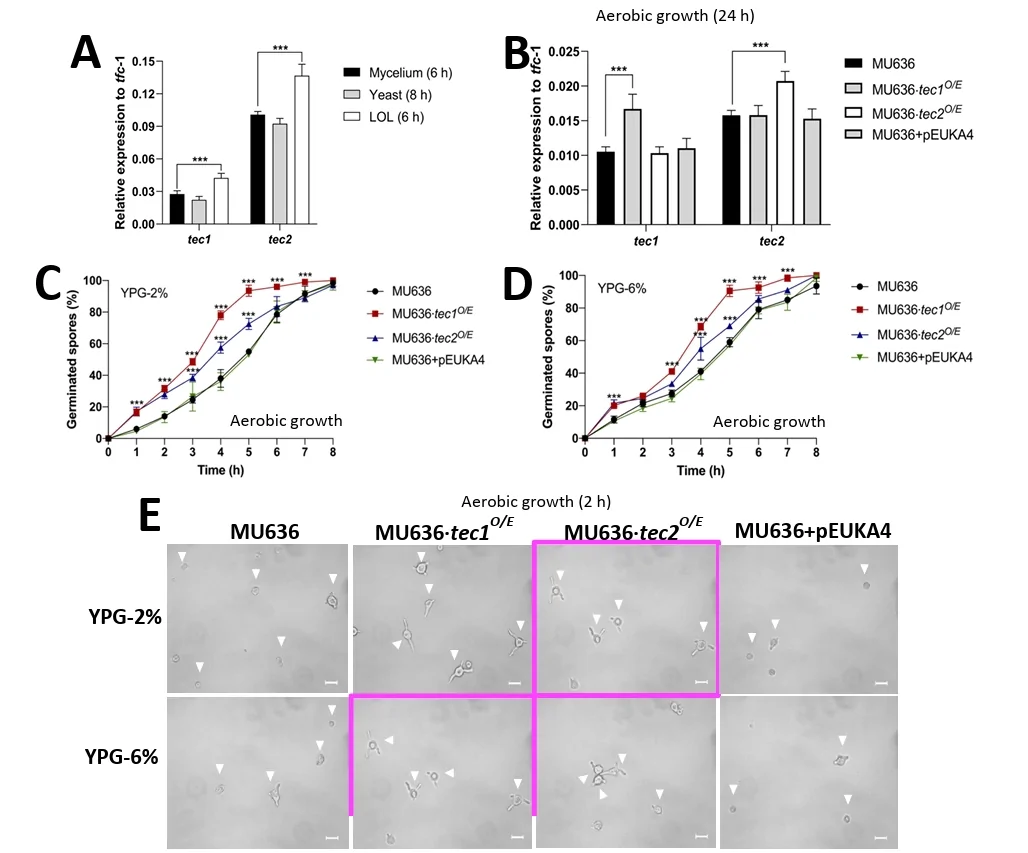



**New figure 1**

You can see the correct photo in a green box for Figure 1 (New Figure 1).

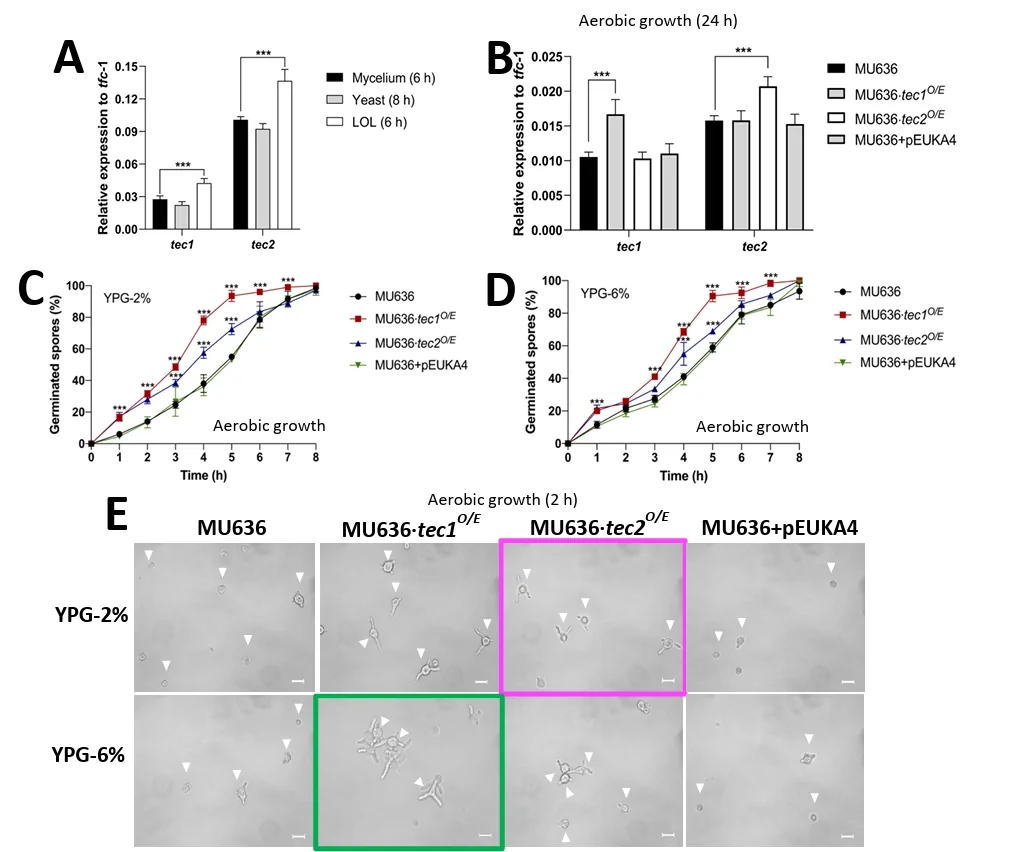



­

**Published figures 2 and 5**

You can see the photos duplicated by mistake in a red box (Figures 2 and 5).

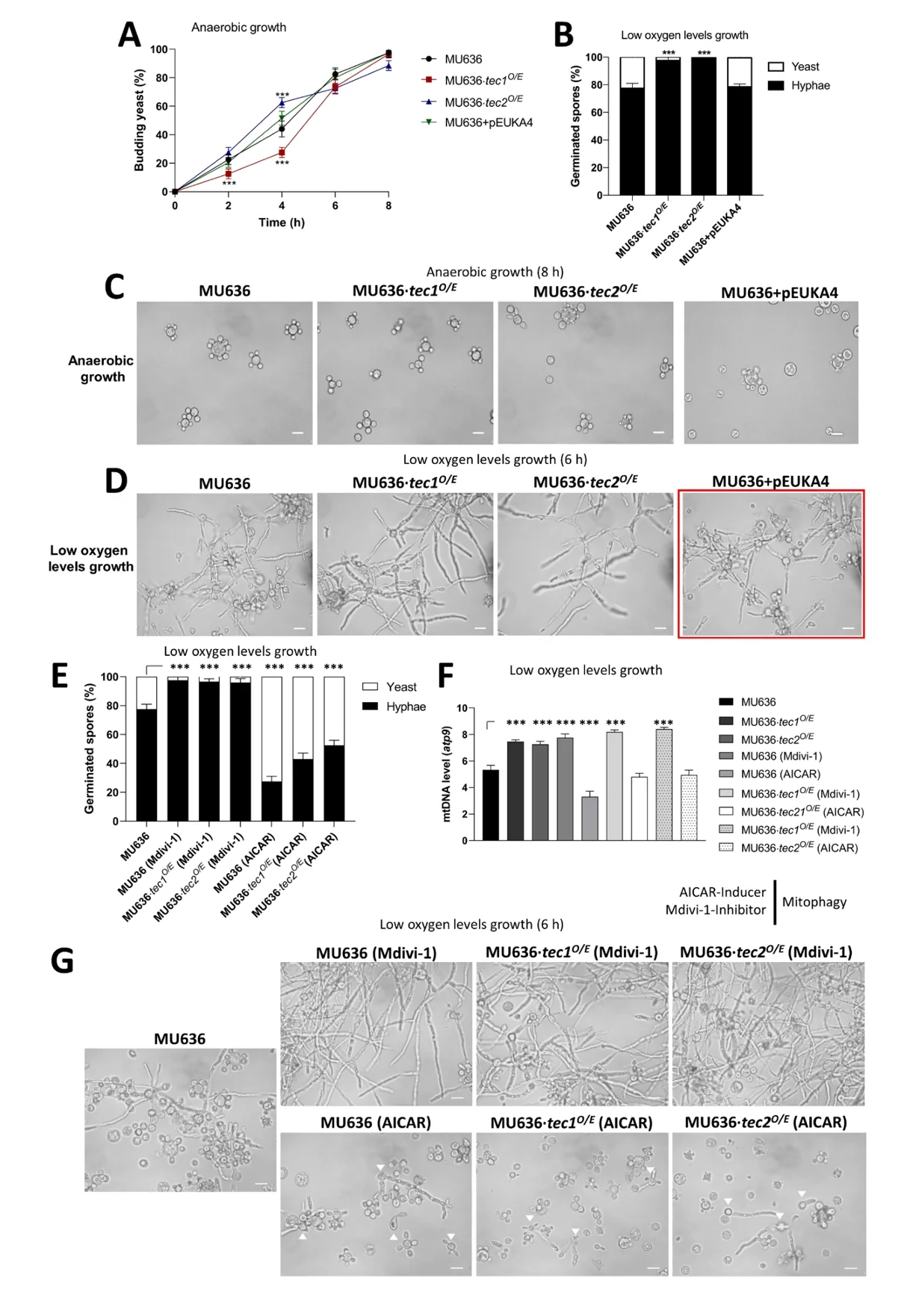

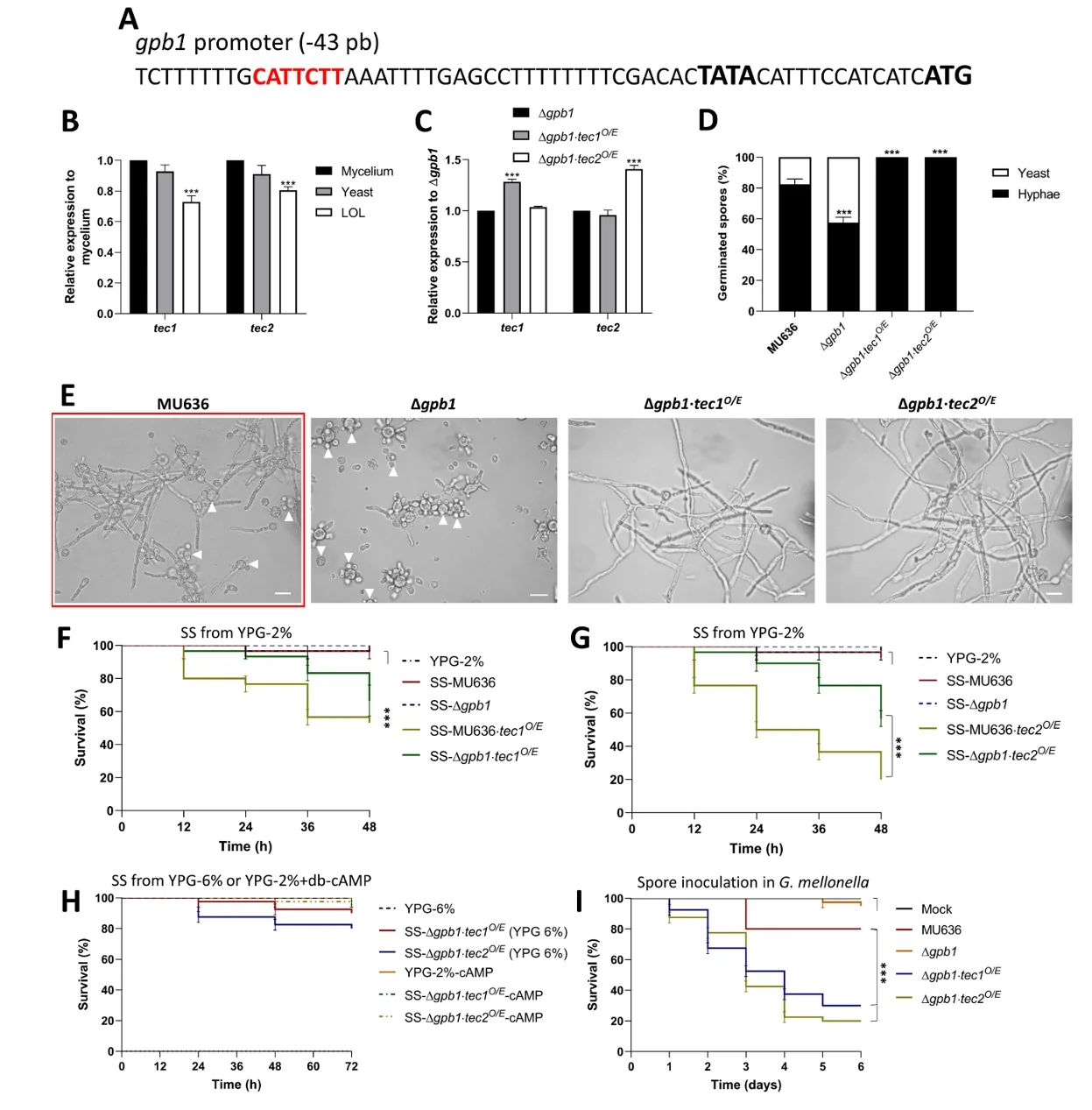





**New figure 5**

You can see the correct photo in a blue box for Figure 5 (New Figure 5).



